# Investigating the Global Dispersal of Chickens in Prehistory Using Ancient Mitochondrial DNA Signatures

**DOI:** 10.1371/journal.pone.0039171

**Published:** 2012-07-25

**Authors:** Alice A. Storey, J. Stephen Athens, David Bryant, Mike Carson, Kitty Emery, Susan deFrance, Charles Higham, Leon Huynen, Michiko Intoh, Sharyn Jones, Patrick V. Kirch, Thegn Ladefoged, Patrick McCoy, Arturo Morales-Muñiz, Daniel Quiroz, Elizabeth Reitz, Judith Robins, Richard Walter, Elizabeth Matisoo-Smith

**Affiliations:** 1 Department of Archaeology and Palaeoanthropology, University of New England, Armidale, Australia; 2 International Archaeological Research Institute, Inc., Honolulu, Hawai‘i, United States of America; 3 Department of Mathematics and Statistics, University of Otago, Dunedin, New Zealand; 4 Micronesian Area Research Center (MARC), University of Guam, Mangilao, Guam, United States of America; 5 Florida Museum of Natural History, University of Florida, Gainesville, Florida, United States of America; 6 Department of Anthropology, University of Florida, Gainesville, Florida, United States of America; 7 Department of Anthropology, University of Otago, Dunedin, New Zealand; 8 Australian Rivers Institute, School of Environment, Griffith University, Nathan, Queensland, Australia; 9 National Museum of Ethnology, Osaka, Japan; 10 Department of Anthropology, University of Alabama at Birmingham, Birmingham, Alabama, United States of America; 11 Departments of Anthropology and Integrative Biology, University of California, Berkeley, California, United States of America; 12 Department of Anthropology, University of Auckland, Auckland, New Zealand; 13 Pacific Consulting Services, Inc., Honolulu, Hawai’i, United States of America; 14 Depto. Biologia, Universidad Autónoma de Madrid, Madrid, Spain; 15 Dirección de Bibliotecas, Archivos y Museos-Proyecto Fondecyt, Santiago, Chile; 16 Georgia Museum of Natural History, University of Georgia, Athens, Georgia, United States of America; 17 School of Biological Sciences and Department of Anthropology, University of Auckland, Auckland, New Zealand; 18 Department of Anatomy and Structural Biology, Otago School of Medical Sciences, and Allan Wilson Centre for Molecular Ecology and Evolution, University of Otago, Dunedin, New Zealand; University of Utah, United States of America

## Abstract

Data from morphology, linguistics, history, and archaeology have all been used to trace the dispersal of chickens from Asian domestication centers to their current global distribution. Each provides a unique perspective which can aid in the reconstruction of prehistory. This study expands on previous investigations by adding a temporal component from ancient DNA and, in some cases, direct dating of bones of individual chickens from a variety of sites in Europe, the Pacific, and the Americas. The results from the ancient DNA analyses of forty-eight archaeologically derived chicken bones provide support for archaeological hypotheses about the prehistoric human transport of chickens. Haplogroup E mtDNA signatures have been amplified from directly dated samples originating in Europe at 1000 B.P. and in the Pacific at 3000 B.P. indicating multiple prehistoric dispersals from a single Asian centre. These two dispersal pathways converged in the Americas where chickens were introduced both by Polynesians and later by Europeans. The results of this study also highlight the inappropriate application of the small stretch of D-loop, traditionally amplified for use in phylogenetic studies, to understanding discrete episodes of chicken translocation in the past. The results of this study lead to the proposal of four hypotheses which will require further scrutiny and rigorous future testing.

## Introduction

Beginning at least 5,400 years ago [1] the chicken (*Gallus gallus*) was domesticated through the purposeful segregation and taming of a few individuals acquired from wild Junglefowl populations in Southeast Asia. Domestication of the fowl is thought, based on archaeological and historical evidence, to have occurred in multiple, independent centers. Chickens were likely domesticated from wild Red Junglefowl [2]–[4], though some have suggested possible genetic contributions from other Junglefowl species [5], [6]. The cultural and religious significance of chickens has contributed to their global distribution [7] and descendants of early domestic fowl have been dispersed around the globe in overlapping waves and by multiple agents over at least two millennia. Chickens are not a migratory species [8], have a small home range [9], do not fly well over long distances [10], and are not equipped for swimming in that they lack webbed feet and glands for the production of water proofing oil. As a result, their current global distribution can be largely attributed to human mediated dispersals.

Understanding when chickens were transported out of domestication centers and the directions in which they were moved provides information about prehistoric human migration, trade routes, and cross cultural diffusion. Possible interactions may be reconstructed by mapping the presence of chickens in archaeological assemblages [11], using historical evidence [12], [13], and perhaps also through the critical application of relationships revealed by mtDNA phylogenies [14]. However, attempts to investigate domestication and dispersal using mtDNA data from modern chickens have been confused by the tangled phylogenies which reflect millennia of overlapping dispersals and over a century of interbreeding for both commercial lines and show breeds. Modern commercial poultry operations produce more than 40 billion birds annually [15] and these are widely distributed around the globe. Therefore, only ancient DNA provides a unit of analysis with the chronological control necessary to reconstruct and disentangle the signals of initial dispersals from later historic interactions, particularly in species that have been the subject of both historic and contemporary crossbreeding.

The origin and domestication of chickens has been of interest to people since at least Roman times [16], [17]. In AD 1600, Ulisse Aldrovandi wrote the first known text focused exclusively on the history and varieties of domestic chicken [18]. Subsequently, using classical texts, passages from the Bible, as well as art and artifacts depicting chickens, scholars traced primary chicken domestication centres and routes of dispersal. These avenues of research led historians to identify centres of domestication in India [19], [20], Malaysia [4], and Burma [12], [21].

Historical reconstructions assist in identifying potential domestication centres and human-mediated trade, but lose resolution in places and eras for which written records do not exist. Archaeological evidence may be used to confirm or refute historical reconstructions and to offer evidence where no recorded history for chickens exists. Archaeological research has identified centres of chicken domestication in India and China; both within the natural range of wild Junglefowl [21], [22]. The oldest *G. gallus* remains have been recovered from 12,000 year old deposits at Nanzhuang in Northern China but, due to their size, the bones are not considered to represent domesticated forms [1]. The earliest undisputed domestic chicken remains are bones associated with a date of approximately 5400 BC from the Chishan site, in the Hebei province of China [1]. In the Ganges region of India Red Junglefowl were being exploited by humans as early as 7,000 years ago [23]. No domestic chickens older than 4,000 years have been identified in the Indus Valley, and the antiquity of chickens recovered from excavations at Mohenjodaro is still debated [24], [25]. Little archaeological evidence is available for early agricultural periods in Burma, Malaysia, and Thailand [26] so it is unclear if independent domestication centres will also be identified in these regions as research progresses.

The distribution of chickens from Asian domestication centers through the Middle East and Europe has been traced along two distinct routes of dispersal using historical, archaeological, and morphological evidence [12], [19], [22]. If these reconstructions are correct then at least two distinct domestication centers contributed chickens to ancient European flocks. However, the genetic signature expected in any specific locale west of Asia will be dependent on the route of introduction, regional trade and exchange relationships, and the effects of colonizing groups. An example of one such group is the Romans who expanded their Empires across Europe, resulting in secondary and tertiary dispersals of a variety of domesticated plants and animals, including chickens [27], [28].

Sufficient archaeological evidence has not yet been compiled to confirm or refute the routes reconstructed by historians. While information regarding the density and distribution of chickens in European archaeological sites likely exists in a number of excavation reports and international publications, it has yet to be fully compiled as has been done for the Pacific [11] and Roman Britain [29]. Currently the best summaries of global distributions can be found in West and Zhou [25] and Serjeantson [27].

In addition to the westward spread to Europe, chickens were also transported eastwards to Island Southeast Asia and subsequently into the Pacific. Preserved chicken remains from archaeological sites in Island Southeast Asia are scarce and the utilization of these birds by ancient humans is more often implied by their depictions on pottery or in paintings than is substantiated by their presence in archaeological sites [30]. However, it has been hypothesized, based on linguistic evidence, that chickens may have been imported to the region as early as 4500 B.P. [30]. The prehistoric distribution of chickens in Oceania is well attested in the archaeological record [11]. In the Pacific, phylogenetic studies of rats [31], dogs [32], pigs [33], and chickens [34], [35] have been used to infer routes of human migration and interaction. The data from these studies strongly suggest that distinct populations of animals were moved into the Pacific at different times and perhaps via different routes [34], [36].

The domestic chicken was dispersed to the Americas, by multiple agents from disparate locations, long after its initial domestication. A Polynesian origin for pre-Columbian chickens recovered from the archaeological site of El Arenal in Chile has recently been proposed [34]. While this has been questioned by some [37], [38] the facts including isotopic information and further radiocarbon dates have been clarified in several subsequent publications affirming the original findings [39]–[41]. European introductions of domestic chickens from Europe and Asia to the mainland of the Americas are well documented after A.D. 1500 [40], [42]. In addition, chickens were brought to the Americas from Africa in the 16^th^ century as a result of the Dutch and Portuguese slave trade [43]. Through the extension of the migratory and exchange networks that carried the descendants of ancient Asian maternal lines of chickens both east and west in prehistory the descendants of the primary Asian lineage converged in the Americas in the post-contact era.

**Figure 1:**
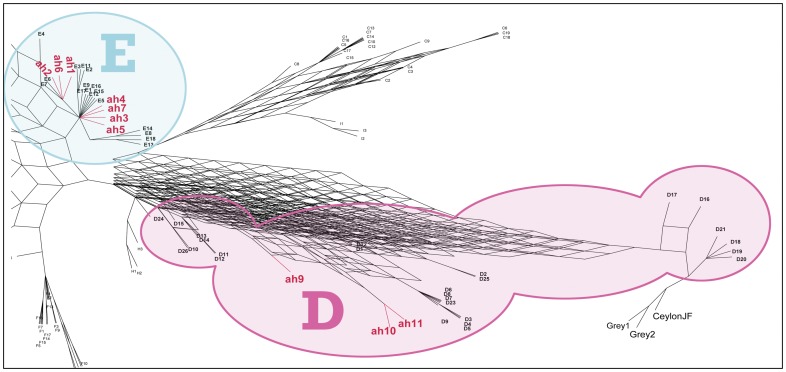
A close up of the E and D branches of a Maximum Parsimony Network showing the affinities of ten of the eleven, non-continuiously numbered, ancient haplogroups detected in our 48 samples with those previously defined by Liu et al. [**14**]. Ancient haplotypes are identified in red bold text and occur in haplogroups D and E. The full network showing the B branch is available as Figure S1.

In the past decade, researchers have focused on the use of specific genetic markers, particularly the control region of mtDNA, as a means of locating individual domestication centers and the routes of subsequent dispersals [14], [44]. Liu et al. [14] have defined nine chicken mtDNA hapologroups encompassing 169 individual haplotypes based on 539 base pairs of the mitochondrial control region. These modern chicken mtDNA sequences may be used to classify ancient sequences in a globally relevant way (see Figure 1 and Table S1).

The use of genetic data to identify centers of origin is based in the study of phylogeography, the underlying assumption of which is that modern samples should show some continuity with ancient samples from a similar geographic location. For these types of studies mtDNA is commonly used as female lines are expected to have more geographic inertia [45]. However, the geographic associations of domesticate haplogroups may also be obscured through trade and exchange. If, for example, chickens from one domestication centre were traded to another region in which Red Junglefowl naturally occurred then domestic individuals that become feral or mixed with native populations might introduce a geographically distinct signature into a wild population. This would interfere with reconstructions of domestication centres using both domestic and wild populations. Given that, in the case of chickens, females are as likely to be transported as males and that introgression events between village and wild Junglefowl are common [21] this may obscure or overwrite the initial mtDNA signature of a group of animals in prehistory.

Phylogeographic analyses comparing ancient and modern chicken sequences have limited utility due to issues related to sampling and human behavior. As of March 2011 modern chicken mtDNA sequences deposited in Genbank (n = 2118), in which the geographic origins were actually documented, were dominated by samples from China (∼37%) and lack a significant cohort of sequences from other regions of Southeast Asia such as India (∼11.7%), Vietnam (∼7.3%), Korea (∼2.4%), Thailand (∼1.2%), and Burma (∼0.5%). Recent investigations have shown that multiple mtDNA signatures of ancestral haplogroups are present in contemporary flocks which live within the natural range of Junglefowl and in areas, such as India, with high potential as ancient domestication centers [44]. This modern mix of haplogroups in Indian fowl is no surprise given the history of interaction between major domestication centres in East and Southeast Asia. The first documented movement of a chicken between two domestication centres was in 1400 BC when Chinese monks brought a chicken home from India [12]. As a result, it is not yet possible to associate specific haplogroups identified in modern chickens with definitive ancient domestication centers. Additionally a number of known historic processes, including the extensive export of chickens from China for the development of show breeds in the 1800s [22], leave the conclusions reached using only modern chicken DNA data in doubt. Due to these complicating factors we obtained archaeologically associated chicken bone samples from several regions of the world to investigate the potential for ancient DNA to contribute to the reconstruction of early global dispersal events involving domestic chickens.

## Results

In total 92 archaeological chicken bones were made available for ancient DNA analysis between 2005 and 2009 (see Table S2). Sequence data were obtained from 48 of the samples. These included: 31 samples from the Pacific (including the Southeast Solomon Islands, Federated States of Micronesia, Vanuatu, Tonga, Samoa, Niue, Hawai’i, and Easter Island); two samples from Thailand; five samples from medieval Spain; three samples from a pre-Columbian site in Chile; and seven samples from the early historic period in the Americas. In order to classify the sequences in a universally relevant way they were named using the established haplogroups defined by Liu et al. [14]. Three haplogroups were detected in the ancient remains. One sample was assigned to haplogroup B, 17 to haplogroup D and 30 belonged to haplogroup E (See Table S3).

In general the archaeological provenience of samples was used as the main criteria for assigning their age. Preference was always given to samples with well defined and clear stratigraphic associations which can be found in the publications referred to in Table S1 and Citations S1. Due to the wide variety of samples and archaeological contexts it is not possible to list the specifics for each sample in this paper. Direct radiocarbon dating and isotope analyses were also requested for individual samples where the context was unclear, in doubt, or where direct radiocarbon dates would assist with the interpretation of results. Samples were sent to either the Rafter Radiocarbon Facility or the University of Waikato Radiocarbon Facility, both in New Zealand, for AMS dating. Dates were considered with respect to the stable isotope values to examine whether a dietary correction was required for their interpretation. Only two new dates are presented for this paper; the dates and isotopes for Vanuatu and Chile have been discussed previously [34], [35], [39], [41]. The dates for the Spanish samples, ESPLCT001 and ESPALB001 were undertaken to confirm the ages provided by AM-M based on context and these dates were as expected with consideration for the contextually associated material culture at each site. Dates determined directly from chicken bones can be found in Table S3.

### Ancient Thai Chickens

Asia, broadly defined, is the geographic area in which one or more domestication events specifically targeting the Red Junglefowl occurred [22]. As a result, ancient chicken bone samples from Southeast Asia will form the basis for modeling the dispersal of chickens from domestication centers to the Middle East and Europe to the west and Southeast Asia and Oceania to the east. Thailand, an area which is within the natural range of Junglefowl, has previously been identified as a domestication centre [3]. The earliest *G. gallus* samples to be identified in Thai archaeological contexts are dated to ca. 4000 B.P. [46]. The cultural importance of chickens in the region is highlighted by the intentional interment of chickens with human burials in the archaeological sites of Non Nok Tha and Ban Na Di [46].

**Figure 2:**
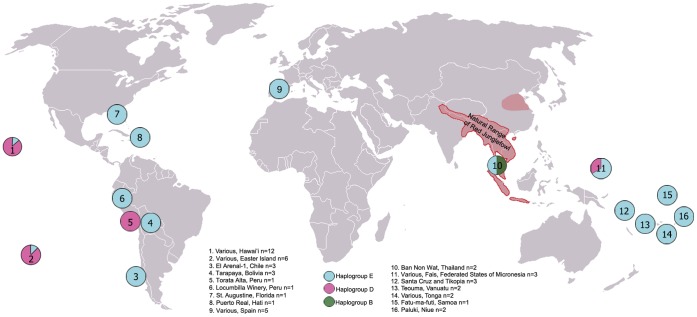
Map showing the relative proportions of haplogroups sequenced from archaeologically derived remains. Each pie represents 100% of the sequences obtained and the numbers inside each pie refer to the legend which details the geographic provenience and the number of samples from each area. Each colour represents one of three distinct haplogroups. The natural range of Red Junglefowl is outlined in red and represents the area in which initial domestication events must have occurred [8], [21]. The red shaded area in northern China represents an area in which *G. gallus* bones have been recovered from archaeological sites older than 5000 BC. This has led to debate about whether the natural range of Red Junglefowl in prehistory extended further north [13], [22], [25].

Of the ten Thai chicken samples available for ancient DNA (aDNA) analyses from the site of Ban Non Wat, two produced reliable, repeatable sequences. These were assigned to haplogroups B (ah11) and E (ah2/E6) (See Table S1 for information on samples). The B haplogroup sample was associated with archaeological deposits dating to around 2500 B.P. and the haplogroup E sequence was associated with a date of around 1550 B.P. The E sequence was identical to sequences previously identified in archaeological remains from Vanuatu [35], Tonga, and Chile [34] (See Figure 2 and Table S1).

### Ancient Spanish Chickens

Chickens are thought to have initially been transported to Iberia (Spain, Portugal, Andorra, and Gibraltar) by Phoenician traders in the first millennium B.C. [47]. Chicken bones from several Spanish archaeological assemblages representing both Moslem and Christian occupations and ranging in age from 1450 to 450 B.P. were used for ancient DNA analyses. Samples ESPALB002, ESPLCT001, and ESPVAL001, all of which date to a period after 1000 B.P., produced ancient DNA sequences that were categorized as haplotype ah3 in this study and are equivalent to Liu’s E1. Two other samples, ESPALB001 and ESPBUZ002, which dated to 1000 and 1450 B.P. respectively, were also of the E haplogroup but with distinct haplotypes (ah4 and ah7) not identified in the Liu et al. [14] study.

### Prehistoric Pacific Chickens

Archaeological evidence suggests chickens were first transported into the Pacific by Lapita peoples moving eastward into Remote Oceania at least as early as 3000 B.P. [11]. This persistent eastward expansion resulted in the translocation of chickens from sites in the Reef and Santa Cruz Islands in the southeast Solomon Chain to Central Eastern Polynesia, and ultimately out to the extremes of the Polynesian triangle including Hawai’i and Easter Island [11]. Ancient DNA analyses of archaeologically associated Pacific chickens revealed two major haplogroups, D and E. Of the thirty-one Pacific mtDNA samples sequenced to date, fifteen belong to haplogroup E and sixteen to haplogroup D. The temporal distribution of the haplogroups is uneven and this led to questions about whether or not the introduction of the D and E haplogroups was contemporaneous and by what route or routes they were introduced.

Of the fifteen haplogroup E individuals in the Pacific, twelve were either archaeologically associated with or directly dated to a period before 1000 B.P. These include three of the earliest animal remains from which mtDNA has been obtained in the Pacific: two bones from Vanuatu, both of which have been directly dated [35] and one bone from the site of Mdailu in the southeast Solomon Islands. The earliest of the samples from Vanuatu has a two sigma calibrated radiocarbon date of 3250–2950 cal B.P. and is directly associated with a Lapita era burial from the Teouma site [35]. The sequence from the archaeological site at Mdailu was derived from a chicken bone which was recovered in association with decorated Lapita ceramics at the site SE-SZ-33 [48]. Haplogroup E has also been detected in other early, post-Lapita era samples predating 1000 B.P. from Tikopia (n = 2), Fais (n = 2), Vanuatu (n = 1), Tonga (n = 2), and Niue (n = 2). In addition, one sample from Samoa dating to between 1000 and 500 B.P. as well as two later prehistoric samples from Hawai’i and one sample from Easter Island were all of haplogroup E.

Chickens belonging to haplogroup D are represented by one individual from Fais, ten samples from Hawai’i, and five from Easter Island. The earliest ancient chicken bone which has a haplogroup D signature is from Easter Island and is associated with a 2σ age range of 660–520 cal B.P. This indicates that the D type chickens had to have been transported into the Pacific at some point prior to 700 B.P. in order for them to have been dispersed this far east by this early date.

### Early Dispersals of Chickens to the New World

Samples from early post-contact period sites in Bolivia, Peru, Haiti, and Florida produced mtDNA sequences of two haplogroups, D and E. The E haplogroup samples were recovered from archaeological sites associated with Spanish colonial forays both east (Haiti and Florida) and west (Bolivia and Peru). Not surprisingly chickens introduced to Florida and Haiti in the 1500s and 1600s have the same signature (ah3/E1) as chicken bones from Iberian archaeological sites dating to between A.D. 1000 and 1500. However, to date, E type samples from 17^th^ Century deposits in Bolivia and Peru represent unique haplotypes from those in Spain and the Pacific. These New World haplotypes were designated as ah5 and ah6 as they have no Liu et al. [14] haplotype equivalents. Finally, all three samples analyzed from the prehistoric coastal archaeological site of El Arenal in Chile also belong to haplogroup E [34]. One haplogroup D sequence was obtained from a Peruvian sample which was excavated from an archaeological site dating to an occupation during the late 1500s/early 1600s [49].

## Discussion

The observed geographic and temporal distribution of ancient mitochondrial haplotypes and haplogroups led to the formulation of several hypotheses for further testing. These are discussed in this section along with suggestions about the potential for nuclear DNA to address questions of migration, interaction, and the origins of domestic lines not suited to the analysis of the mtDNA control region alone.

### Hypothesis One: Temporally Distinct Introductions of Chickens to the Pacific

The samples from the island of Fais, located in the Federated States of Micronesia, provide a temporal component to the analysis of Oceanic chickens as individual bones and their DNA sequences represent distinct temporal periods. The two samples which come from archaeological contexts predating 1000 B.P. both belong to haplogroup E, while the later sample, which is contextually associated with a charcoal date of 600±40 B.P. (660–530 cal B.P.) [Beta-286414] belongs to haplogroup D.

Prehistoric interactions between northern Melanesia and the Caroline Islands [50] could have easily included the transfer of chickens from the Solomon Islands and/or Vanuatu to Micronesia. Fais, a small island, utilized wide trade and exchange networks to safe guard against environmental failures or weather related catastrophes. This is reflected in the archaeological assemblage which has been found to contain steady levels of imported materials [51], highlighting the importance of cultural contacts, particularly with Yap. Archaeological excavations in Fais have revealed that in Level IV deposits new items appear, including laminated potsherds and *Cassis* sp. scrapers, which likely reflect changes occurring in Yap [52]. These artifactual changes may signal the arrival of a new group of people, or new cultural influences in the region. These new items also appear to coincide with the temporally distinct appearance of haplogroup D chickens after 660 B.P. [52]. While more research and a larger cohort of samples is necessary to investigate the temporal division observed in the chicken DNA signatures, this is not the first paper to highlight the importance of Micronesia in the dispersal of plants and animals within Oceania [53]–[55].

If haplogroup D chickens were introduced to the Pacific later than those belonging to haplogroup E, the two lineages may have converged before they were dispersed, as a polymorphic population, to Hawai’i and Easter Island. Both haplogroups appear in early period archaeological sites in East Polynesia. Unfortunately no samples from later prehistoric periods in Tonga and Samoa have resulted in sequence data to test this hypothesis. In addition, no samples have yet become available from faunal collections in Central Eastern Polynesia. Samples from these archipelagos and from later periods of prehistory will be required to test the hypothesis for temporally distinct introductions of chickens to the Pacific in prehistory. Unfortunately, the paucity of chicken remains in Near Oceanic archaeological sites and debates about the existence of prehistoric chickens in the Mariana Islands, particularly Guam [11], [56], make identification of probable dispersal routes and their chronology very difficult at present.

### Hypothesis Two: The Appearance of Haplogroup D in Peru may Reflect a Pre-Columbian Introduction

Haplogroup D has not yet been detected in any ancient chicken bone samples from Europe or from Thailand. Thus far it has only been identified in ancient Polynesian and Micronesian chicken remains as well as a single Peruvian sample. The available sample size for this study is too small to be representative of global chicken mtDNA diversity in prehistory and offers only a preliminary glimpse upon which to build future studies. However, the identification of haplogroup D in early post-contact deposits in Peru is tantalizing and requires further consideration.

The first documented introduction of chickens to Peru was by Alonso de Molia to the city of Tumbes via Panama in AD 1528 [57]. The Manila galleon trade was well established by the late 1500s linking the west coast of South America, the Mariana Islands, and the Philippines via Island Southeast Asia [58]. Records exist for trade of foodstuffs including chickens in the Marianas as early as A.D. 1581 [59]. However, the existence of prehistoric chickens in the Marianas is highly debated [56], [60] and they are not noted as present in the earliest European reports of Guam [59], [61].

The early date of the Peruvian assemblage from which this haplogroup D sample was recovered raises the possibility that it could represent a descendant of a chicken haplogroup introduced from Polynesia. The fact that the sequence from the chicken bone from the Torata Alta site in Peru is identical to one from Fais in Micronesia also may support a possible Pacific connection.

Future studies would benefit from examining a larger cohort of chickens from the Torata Alta site and also from other sites nearby in an attempt to determine if this does represent a link to a prehistoric Polynesian haplogroup and if so whether it was a prehistoric or a post-Columbian introduction. At present this lone haplogroup D American chicken is as likely to be the result of early Spanish forays into Oceania and Southeast Asia as it is evidence for pre-Columbian contact with Polynesia. However, pre-Columbian contacts between Peru and the Pacific are suggested by independent lines of evidence including the prehistoric transport of sweet potatoes from South America to Oceania [62] and similarities in the terminology for this domesticate in Quechuan and a variety of Polynesian languages [63]. Simulated voyages have supported the likely landfall of Polynesian voyagers in the region of Ecuador and Peru [64].

### Hypothesis Three: Identification of a Definitive mtDNA Signature(s) for a Thai domestication Centre in Doubt

One of the early studies of chicken mtDNA signatures concluded that all domestic fowl were descended from ancient Thai hens [2]. Unfortunately the results of the current ancient DNA study are not sufficient to identify a Thai domestication centre. Aside from obvious issues with the small sample size, there are problems relating to millennia of documented trade and exchange relationships between Thailand, India, and China [65], [66], which are likely to have involved chickens as well as other domesticated animals and plants. This includes a well known proclivity for Chinese breeders of fighting roosters to regularly import stocks from Thailand and other areas of Southeast Asia to improve lines [67]. While this likely focused on trade in roosters it also increased the probability of hens crossing geographic boundaries. However, the existence of haplogroup E in an ancient Thai sample does demonstrate that the lineage was present in ancient Southeast Asia and thus was available to be dispersed both to Europe via the Middle East and to the Pacific via Island Southeast Asia by at least 1550 B.P. and possibly much earlier. The ultimate geographic origins of haplogroup E remain to be confidently established and its relationship to Thailand may be difficult to adequately define.

### Hypothesis Four: Low mtDNA Diversity for Chickens, Ancient and Modern, has Serious Consequences for the Utility of mtDNA Alone in the Reconstruction of Prehistoric Events

One of the more striking results of this study was the discovery that the same mtDNA haplotype (ah3) was present in ancient chickens derived from archaeological sites in Europe, Thailand, the Pacific, and Chile from samples spanning thousands of years. As there is no evidence for Spanish incursions into Remote Oceania before A.D. 1521 (429 B.P.), the existence of identical authentic mtDNA sequences in bones derived from post-1000 B.P. Spanish sites and those from securely prehistoric contexts in the Pacific and Chile point to an ancestral node representing a single ancient domestication centre in Asia.

No chicken DNA amplicons have ever appeared in PCR blanks or negative extractions, during ancient chicken extractions in two separate laboratories, and over a period of four years making it highly unlikely to represent contamination – as has been suggested in the past [37]. The first ah3 (Liu et al.’s [14] E1) sequence was observed in sample HWAKUA001 in 2006, with the final sequence of SLB33001 that was generated in 2009. Amplicons longer than 175 bp were rarely obtained for older specimens representing haplogroup E. In fact attempts to extend the sequence by using overlapping primer sets to generate sequence of 250 bp per amplicon were unsuccessful in all but the most recent specimens. This is appropriate molecular behavior for ancient DNA [68] as one expects that contaminating modern DNA would amplify comparatively easily and produce longer amplicons. In addition the detection of identical haplotypes in extant animals and ancient samples has also been reported for both Pacific rats [31] and pigs [33].

The pattern of a single geographically dispersed haplogroup signature with several smaller sub-clusters has been reported in previous studies of the phylogeography of chicken mtDNA by several groups of researchers [14], [37], [44]. This mimics patterns observed for goats [69], sheep [70], and dogs [71], [72]. These relationships likely reflect the complex history of human-mediated translocation of these animals. The study of short regions of the mtDNA control region of modern chickens suggest that this region is not sufficiently variable to act as more than a broad phylogenetic marker for the dispersal of the domestic fowl in prehistory.

In the 1990s studies of mtDNA sequences led to widespread concern that chickens, particularly commercial breeds, were highly homogenous and that immediate conservation steps were required to preserve the remaining diversity [73]. However, researchers studying aspects of nuclear DNA diversity, even simply in terms of SNPs, suggest there is a great deal of diversity in modern chicken populations [15]. This not only supports our hypothesis but strongly suggests that a combination of full mitochondrial genomes and select nuclear DNA markers will be required to build more sophisticated models of prehistoric dispersals of domesticated chickens.

The available evidence may also indicate that if haplotypes cannot distinguish between widely separated contemporary populations (e.g. ancient samples from Spain and Polynesia), that modern mtDNA is largely unsuitable for use in the identification of human mediated transfers in prehistory [74]. This may be due, in part, to a preferential transport of hens rather than roosters. Several scholars have postulated that the purpose of chicken domestication was cockfighting [13], [75]. However, it has also been proposed based on comparative morphology, historical depictions, and genetic relatedness that egg type chickens are the most ancient breed [76], [77]. The protein conversion from animal feed to food source is highly efficient in the production of eggs, second only to milk [27]. This may have been an attractive feature of domestic chickens, which were also highly portable producers of these secondary protein sources. This would encourage the transport of females perhaps even to the exclusion of males. Roosters are only required for the fertilization of eggs, not their production.

Not only are eggs a ready source of fresh protein they have also been used in wine making, in medicine, as binding agents for pigments, as hair products and in ritual [18], [78]. In fact the frequent inclusion of chickens and eggs in Roman burials led some to speculate it was the ritual importance of chickens that led to their initial transport out of Asian centers [27], [79]. Therefore, the phylogeographic assumption that females have greater geographic inertia may be violated in the study of chickens by the widespread use of eggs as a dependable protein source, and in some cases as a monetary unit. In both the Americas [43] and the Philippines [80] the use of eggs as tribute is well documented.

It is only in situations where cock-fighting was the primary motivation for the breeding and dispersal of chickens that the traditional phylogeographic associations about the geographic inertia of females are likely to hold [45]. The purpose of specific groups of animals therefore has an immediate impact on the phylogeographic reconstruction of their ancient history. The literature for the Pacific [81], [82] and Asia [83] reveals people were less likely to trade hens as they were used for breeding stock. And yet their value may also mean that people were more likely to take these prized animals with them when they moved or would have traded them at a higher exchange rate.

Archaeology may be called upon to sort out the use of chickens in particular places at particular times. Sex ratios in faunal assemblages may indicate sport in archaeological deposits where the bones of males are more frequently recovered than females, though this may also indicate ritualistic use [27]. Fighting cocks are thought to be smaller and leaner and may be distinguished in the archaeological record from food types by the size of their skeletal elements. Laying hens may also be distinguished by a thickening of their medullary bones in preparation for egg production. Another underutilized indicator for the exploitation of eggs in prehistory is the presence of egg shell in archaeological deposits. Currently this line of evidence in inhibited by taphonomy, a lack of targeted collection from archaeological excavations, and subsequent identification [27]. Where it is available the collection of egg shell demonstrates that chicken eggs were important in both Europe and North Africa from at least the middle of the first millennium BC. At this stage given not only the low diversity observed in ancient and modern chicken mtDNA but also the female specific W chromosome [84] it is worth reconsidering the primary assumption that hens should show more geographic inertia than roosters in the global dispersal of chickens. This should be explored using a combination of traditional archaeological analyses and genetic studies.

The unique nutritional and economic traits of hens in combination with their ease of transport are likely to increase the translocation of animals, thereby blurring the geographical boundaries for domestication centers and rendering phylogeographic methods ill equipped to provide the sort of data necessary identify origins [45]. Examples include documented instances of transcontinental chicken dispersals initiated by the Romans in the early first millennium [28], [29], European Explorers from the 1500s onwards [58], [85], and Poultry Fanciers in the 1800s [22], [85]. Indeed historically attested transfers can cause serious issues for the researcher pursuing origins using modern DNA evidence alone [74]. Therefore in order to apply modern DNA data to reconstructing past events one must undertake a great deal of research into specific episodes of chicken dispersal to assess the match between DNA signatures and historical records. This will be complicated by the fact that many historic transfers are not well recorded. At this stage, due to the restriction of current molecular techniques, inadequate mathematical models for the complexity of the human mediated dispersals, and the lack of a coherent review of the history of chicken distribution, the endeavor to reconstruct the past using mtDNA data will depend on a comparative approach. Ancient samples from sites all over the globe representing nodes in well documented trade and exchange routes will allow for the evaluation of prehistoric signals in modern chicken populations and could provide a means by which the ultimate origins of specific haplogroups may be determined. This evidence will require careful consideration with reference to the archaeology and historical evidence.

### How can Future Research Build on These Observations?

This study represents the largest ancient mtDNA dataset for chickens published to date and demonstrates the need for integrated archaeological and genetic programs, as opposed to studies of modern DNA variation alone [74]. Perhaps the most striking result reported here is the evidence that the haplogroup E chickens were taken in opposite directions out of Asia and their histories and dispersal pathways finally converged in the Americas after A.D. 1500, possibly as much as 4000 years after the initial domestication of the lineage from the wild Junglefowl of Asia.

For modern DNA to make a more useful contribution to the study of prehistoric chicken transfers the sampling of chicken populations around the world must be evaluated using a geographically enriched and more balanced datasets. As discussed previously the greatest proportion of modern DNA, originating within the natural range of Junglefowl is from China, with few sequences available from other potential centers such as Burma and Thailand. In addition modern mtDNA sampling tends to focus exclusively on the hypervariable region of the d-loop, ranging in length from 300 to 600 bp of sequence [14], [47], [86], [87]. Using modern techniques and modern materials it is advisable that investigators amplify the entire mtDNA genome which may, perhaps, identify variability in other regions that may be useful for segregating haplogroups and haplotypes. Such markers could then be targeted in ancient samples.

A great deal of information can be generated using modern genetic techniques, however, that for domesticated animals is rarely useful when divorced from history. It is no longer sufficient in applying genetic information to human history to let the sequences do the talking. In order to test the phylogeographic assumptions about continuity of populations and the geographic inertia of females, better documentation is required. Full descriptions of the animals from which sequences were derived, their precise geographic provenience, and an attempt to discuss both breed histories (where appropriate), and the potential for interbreeding between wild and domestic stocks [88], [89] are key to understanding the genetic data. A myriad of data exists on the crossings between breeds of various origins by a range of people in history in order to produce particular results [20], [85], [90], [91]. These are essential pieces of information if only to tell researchers which samples and sequences will not be useful to reconstructing geographic origins. Recent studies have integrated this approach showing more clearly the limitations of using only modern DNA data to reconstruct more ancient events [86], [92]. The findings of this study strongly suggest that phylogeographic studies, based on mtDNA sequences alone are inadequate to reconstruct highly detailed histories of human translocations, and domestication processes.

Translocation and domestication studies may be enhanced through the targeting of key nuclear loci, particularly those connected to changes associated with domestication, that have the potential to separate wild and domestic populations [93]. In addition, the capacity of hens to produce eggs in the absence of roosters may indicate, that in chicken populations, geographic inertia will be associated with males. This may be complicated by the known mechanism of sperm ejection of undesirable mates by hens [94]. If the purpose of transporting chickens is to provide a ready source of protein and roosters are known to exist at the final destination there is no need to transport males at all. However, even nuclear markers will be limited in their phylogeographic utility due to the complex history of domestic animal transfers in both historic and prehistoric times [95]. With new Next Generation Sequencing Technology [96] the opportunity now exists to use ancient nuclear DNA in these endeavors. While modern DNA will be indispensible in the identification of domestic genes to be used in ancient DNA research, it is not useful in isolation to reconstructing episodes which occurred in the prehistoric period.

### Conclusions

As a result of the careful analysis of archaeologically associated, and in some cases directly dated, ancient DNA samples an early global distribution of haplogroup E chickens has been revealed. This dispersal out of Asia began before 3000 years ago and involved the movement of chickens both westwards to Europe and eastwards into the Pacific. The distribution of haplogroup D likely represents a separate dispersal into the Pacific from a distinct Asian domestication centre. The eventual identification of these centers will greatly enrich our understanding of chicken domestication and the history of dispersals from multiple locations. While unambiguous data does not yet exist to trace any of the detected mtDNA signatures back to specific domestication centers, the analysis of ancient DNA sequences presented here is an important first step towards it. Future research needs to focus on markers identified, from both full mtDNA genomes and nuclear genes which are subsequently targeted in ancient specimens, examined within their historical and/or archaeological context.

## Materials and Methods

This project was originally conceived as a study of the mtDNA signatures of ancient Pacific chicken remains to identify prehistoric migration and interaction. All chicken remains excavated from the Pacific which were available for destructive testing were collected for this project and thus are reflected in the disproportionate number of samples from the Pacific in this analysis. After the discovery of a haplogroup E sequence in the pre-Columbian remains from the site of El Arenal in Chile [34] the study was expanded to explore whether the E signature that they shared was indeed unique to the Pacific. This led to the acquisition of samples from sites in the Americas established by the Spanish after 1492 as well as the examination of sequences from Spain securely dated to a period before Columbus for comparison with the Pacific and Chilean samples. Samples from Thailand were targeted as the region had previously been identified as a domestication centre for chickens and thus presented the opportunity to investigate their relationship to the widespread occurrence of haplogroup E individuals in this study.

Ancient DNA extractions and PCR set up were conducted in the Department of Anthropology Ancient DNA facility at the University of Auckland. This Facility follows the standards to monitor for contamination and establish the authenticity of ancient DNA set out by Cooper and Poinar [97] and Pääbo et al. [68]. The ancient DNA laboratory is a physically isolated work area, to which access is carefully controlled and all occupants dress in disposable gowns, wear hairnets, face masks, disposable booties (or shoe covers) as well as latex gloves. No modern chickens have been processed in that laboratory and to avoid contamination with either modern or amplified DNA the workflow is unidirectional with no movement of materials back into the ancient laboratory. People must shower and change their clothing before beginning a new round of extractions in the ancient laboratory. All stages of work from extraction to PCR amplification include negative controls in which no sample or template aliquots have been added. Positive controls were rarely employed but when used were ancient chicken samples of known performance.

Prior to extraction, samples destined for ancient DNA analysis were photographed, weighed, and when appropriate, measured. Samples were prepared by cleaning the adhering soils from the outside and interior surfaces. For the exterior this was accomplished by sanding the outer surface of the bone with sterile sandpaper. A sub-sample of each of the archaeological bones was processed and the remainder stored for future use (such as independent replication and radiocarbon dating). The bone sub-samples were ground using sterile mortars and pestles. Ancient DNA extractions for all archaeological bones were carried out using a modified guanidine thiocyanate silica suspension technique [98]. Blank extraction controls were used with each extraction and negative PCR controls were always employed to detect possible contaminants which may have been present in reagents or labware.

Ancient DNA template molecules were also monitored for appropriate molecular behaviour. Some samples (such as CHLARA001) were run with primer sets designed to amplify longer target molecules (over 300 bp) as a test to determine if the DNA behaved as ancient DNA is expected to; that is that only small products are amplified. Our experimental design included the parameter that amplicons longer than 250–300 bp would be suspect, as they would not meet the authentication criteria, and would therefore be discarded [68]. No sequences of this length were observed during the period of the study despite the fact that in several instances amplification of templates longer than 300 bp was purposefully attempted in order to assess the potential for contamination. Sequences were only considered valid if they were amplified more than once, sequenced in both directions from separate PCR products and the sequences were concordant. In cases of particularly important or special samples independent replication was carried out at a separate ancient DNA facility.

PCR amplifications for ancient DNA products were performed in 30 µL reaction volumes containing 1 unit of ampli*Taq* DNA polymerase (ABI Applied Biosystems), 1× PCR Buffer, 0.15 mM each of dNTPs (Pharmacia), 0.5 µM each primer, 1.0 mg/mL BSA, 2.4 mM MgCl_2_, and 5 µL of target DNA. Samples were then run on a Bio-Rad iCycler Thermal Cycler (Bio-Rab Laboratories Incorporated, California). Initial denaturing was at 94°C for 2 min; 45 cycles followed, each with a denaturing step at 94°C for 20 sec, an annealing step at 54–50°C (depending on the primer pair) for 20 sec, and extension step at 72°C for 20 sec. A final extension step of 5 min at 74°C followed, and samples were then cooled to 15°C. Negative control samples, in which no target DNA was added, were used in all amplifications to check for contamination.

Of the nine major studies of chicken mtDNA affinities published before 2008, seven targeted a segment of the control region shorter than 600 bp [2], [14], [99]–[105]. The eleven most commonly observed SNP sites (by more than two authors) within this region were 167, 237, 243, 256, 261, 281, 301, 306, 310, 330, and 342 (numbered relative to the reference sequence NC_001323). This 175 bp region is an ideal length to target for ancient DNA studies as it contains a great deal of diversity in modern chicken mtDNA. This was accomplished using various combinations of primer pairs to amplify the longest overlapping sequences possible for each sample (Table S4). The number of amplicons which were successfully sequenced for each sample are shown in Table S5.

Amplified PCR products of samples were visualized on a 1∶1 Agarose: Nusieve (2%) gel stained with 2% ethidium bromide, purified in sephacryl columns (Microspin S300, from Amersham, Pharmacea, Biotech) and quantified on 2% ethidium bromide stained agarose gels using a low mass ladder. Direct sequencing of PCR products was carried out at the Allan Wilson Centre for Molecular Ecology and Evolution (Albany Campus Sequencing Facility) using the BigDye™ Terminator Version 3.1 Ready Reaction Cycle Sequencing Kit run using a capillary ABI3730 Genetic Analyzer, from Applied Biosystems Inc.

A sub-sample of THABCHO009 was also sent to Massey University in Albany (New Zealand) for independent replication of results, as had previously been done for samples CHLARA001 [34] and VUTTEO003 [35]. At Massey, DNA extraction and amplification were carried out as outlined in Huynen et al. [106]. Amplified products were purified by centrifugation through Sephacryl S200 columns and were cloned into pCR 2.1 (Invitrogen).

### Phylogenetic Analysis

A total of 181 chicken sequences were aligned: including a representative sequence from each of the eleven ancient haplotypes (ah), sequences representing the167 of the 169 haplotypes identified by Liu et al. [14], as well as sequences from two Gray (EU847741 & EU847742) and a Ceylonese Junglefowl (EU199948) using MUSCLE software [107]. The resulting alignment was then manually checked and trimmed to 175 base pairs (bp), the length shared by both the ancient and modern data sets. The haplotypes observed in the ancient samples were named using the abbreviation ah to stand for ancient haplotype. Where these are identical to a sequence previously classified by Liu et al. [14] they are identified using both by an ah# and the published nomenclature. For example ah3 is equivalent to Liu et al.’s E1 [14]. In several cases no Liu equivalent was identified and thus only the major haplogroup to which ancient samples belong can be identified by the ah# designation.

In our comparison with previously published studies, sequences were examined for haplogroup defining Single Nucleotide Polymorphisms (SNPs). 175 base pairs (bp) were compared and over 181 sequences; and 175 unique haplotypes and nine haplogroups were found. The relationships were assessed using Maximum Parsimony (MP) Methods in PAUP* [108], utilizing the Blue Gene Server at the University of Canterbury. This resulted in 168,400 most parsimonious trees which were imported to SplitsTree [109]. Using the 382 splits both a Consensus Tree and a Consensus Network were constructed [110]. Even at the reduced sequence length (175 bp cf 520 bp) haplotypes as defined by Liu et al. [14] maintain their haplogroup affinities (Figures S1 and S2).

### Radiocarbon Dating

For several of the samples, direct radiocarbon dating was undertaken. Samples were sent to one of two labs for dating, The Rafter Radiocarbon Laboratory at GNS Science in Lower Hutt, New Zealand and Waikato Radiocarbon Laboratory at Waikato University, New Zealand. Samples were calibrated using OxCal [111] and the appropriate Hemisphere Curves [112], [113]. Associated isotope values are reported in Table S3.

### Permissions for Use and Context of Archaeological Samples

All chicken bones used in this analysis were obtained with permission from relevant museums and excavating archaeologists. Excavation of faunal materials was undertaken with the knowledge of the appropriate authorities and with permits as required by the laws of the countries in which they were exhumed. Details of these can be found in the reports of excavations and sites that are listed in Table S2 with other details relating to sample provenience.

## Supporting Information

Figure S1 **Maximum Parsimony Network showing the affinities of the ancient haplogroups detected in ancient chicken samples with those previously defined by Liu et al.** [**14**] **.** Ancient haplotypes are identified in red bold text and occur in haplogroups B, D and E.
(TIF)

Figure S2 **Maximum Parsimony Concensus tree produced using the majority tree rule showing the relationships between the ancient haplogroups detected in archaeologically associated chicken samples with those previously defined by Liu et al.** [**14**] **.** Ancient haplotypes are identified in red bold text. The Ceylon Junglefowl has been used as the designated outgroup.
(TIF)

Table S1 **Information relating to the 48 samples which produced ancient DNA sequences.**
(PDF)

Table S2 **Information relating to the 92 samples acquired for this study. Samples highlighted in blue are those for which mtDNA was amplified.**
(PDF)

Table S3 **Results of direct radiocarbon dating of some of the samples used in the ancient DNA analysis. Those marked with a single asterisk were published in 2008** [**41**] **and those with two asterisks in 2010** [**35**] **.**
(DOC)

Table S4 **Primers employed in the amplification of overlapping fragments of short template DNA.**
(DOC)

Table S5 **Number of uniquely derived amplicons for each sample published for the first time in this paper.**
(DOC)

Citations S1 **Supplementary Citations.**
(DOC)
