# Correction: Investigating the Global Dispersal of Chickens in Prehistory Using Ancient Mitochondrial DNA Signatures

**DOI:** 10.1371/journal.pone.0216626

**Published:** 2019-05-02

**Authors:** Alice A. Storey, J. Stephen Athens, David Bryant, Mike Carson, Kitty Emery, Susan deFrance, Charles Higham, Leon Huynen, Michiko Intoh, Sharyn Jones, Patrick V. Kirch, Thegn Ladefoged, Patrick McCoy, Arturo Morales-Muñiz, Daniel Quiroz, Elizabeth Reitz, Judith Robins, Richard Walter, Elizabeth Matisoo-Smith

After publication of this article [1], a concern was raised regarding the fifth sentence in the last paragraph in the Introduction section. That sentence is as follows: “The first documented movement of a chicken between two domestication centres was in 1400 BC when Chinese monks brought a chicken home from India [12].” This information was cited as originating from a 1913 paper by John Peters [2, reference 12 in the original article]. However, the identity of the human agents of transport (i.e. Chinese Monks) was not included in [2] and was taken from another source.

The oft-cited information regarding the transfer of chickens in 1400 BC found in many sources (e.g., [3–7]) is all related directly to work by Charles Darwin [8]. It was in *The Variation of Plants and Animals Under Domestication* (Vol 1) where Darwin stated: “Mr. Birch of the British Museum, has translated for me passages from a Chinese Encyclopaedia published in 1609, but compiled from more ancient documents, in which it is said that fowls are creatures of the West, and were introduced to the East (i.e. China) in a dynasty 1400 B.C. Whatever may be thought of so ancient a date, we see that the Indo-Chinese and Indian regions were formally considered by the Chinese as a source of domestic fowl.”

It appears that later references to this date, typically without attribution to Darwin, sometimes included embellishments. For example, Brown [6] in his book *Poultry Breeding and Production* (Vol 1) wrote “[t]he statement has been made that Buddhist priests passed from India to China about B.C. 1,500, and that they conveyed the fowl, already domesticated in the former Country, to the Celestial Empire.” Significantly, Brown went on to write “[i]t would appear that the first compulsory migration of the fowl was eastward, that is, to China, where, be it noted, the practice of husbandry was anterior by millenniums to that of western nations.”

As a result the fifth sentence in the last paragraph of the Introduction section should be revised to: “The first documented movement of chickens between two domestication centres is asserted to have been 1400 BC [Darwin, C (1868). The variation of animals and plants under domestication.].”

The authors apologize for omitting the attribution specifically related to the identification of Chinese monks in the published article.
