# Supplementary figures and images for: Investigating the Global Dispersal of Chickens in Prehistory Using Ancient Mitochondrial DNA Signatures
Source: PLoS One. 2012 Jul 25;7(7):e39171. doi: 10.1371/journal.pone.0039171 (PMC3405094; doi:10.1371/journal.pone.0039171)

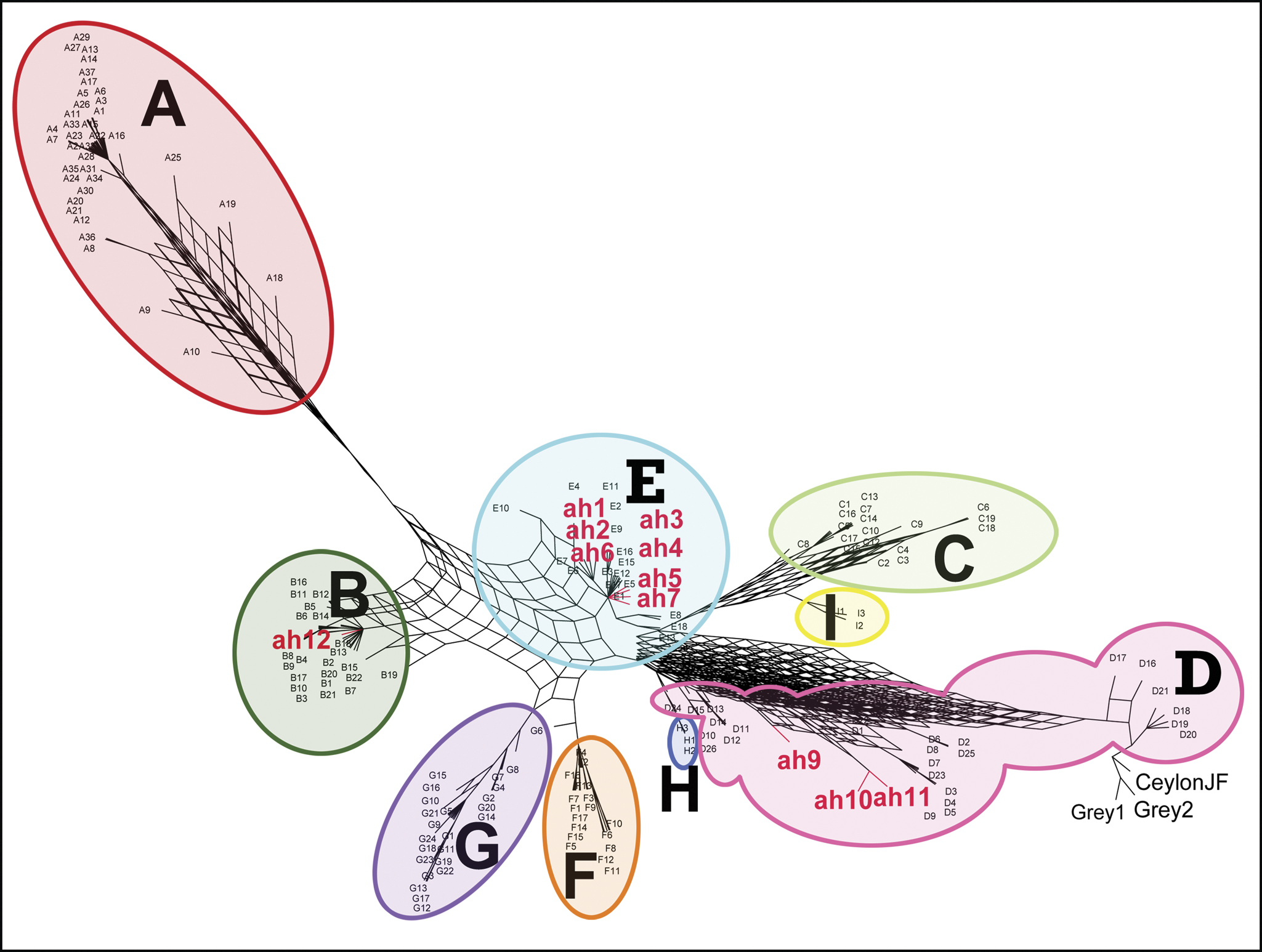

Supplement: Figure S1 — Maximum Parsimony Network showing the affinities of the ancient haplogroups detected in ancient chicken samples with those previously defined by Liu et al. [14] . Ancient haplotypes are identified in red bold text and occur in haplogroups B, D and E. (TIF) [file pone.0039171.s001.tif]

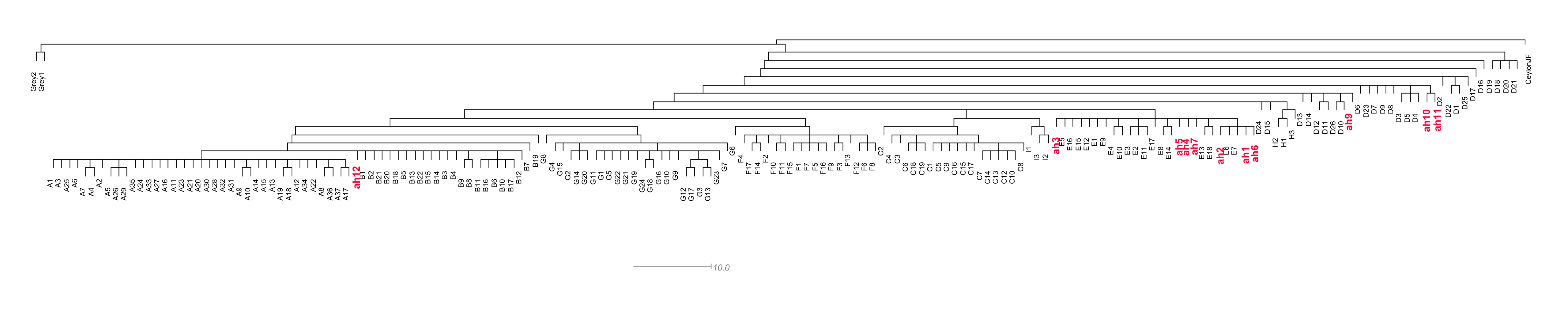

Supplement: Figure S2 — Maximum Parsimony Concensus tree produced using the majority tree rule showing the relationships between the ancient haplogroups detected in archaeologically associated chicken samples with those previously defined by Liu et al. [14] . Ancient haplotypes are identified in red bold text. The Ceylon Junglefowl has been used as the designated outgroup. (TIF) [file pone.0039171.s002.tif]
