# Supplementary material for: Investigating the Global Dispersal of Chickens in Prehistory Using Ancient Mitochondrial DNA Signatures
Source: PLoS One. 2012 Jul 25;7(7):e39171. doi: 10.1371/journal.pone.0039171 (PMC3405094; doi:10.1371/journal.pone.0039171)
Supplement: Table S1 — Information relating to the 48 samples which produced ancient DNA sequences. (PDF) [file pone.0039171.s003.pdf]

Supplementary Table S1: Information relating to the 48 samples which produced ancient DNA sequences.

Haplogroup	Haplotype		Sample Name	Accession Number	Number of Bases Amplified	Reference
	Ancient	Liu Equivilant				
E	ah1		VUTTEO003	HM189678	175	Storey et al. 2010
	ah2	E6	VUTTEO006	HM189679	201	Storey et al. 2010
			Tonga TD	EF535236	183	Storey et al. 2007
			HWIKUA001	EF535238	201	Storey et al. 2007
			NIUPKI009	EF535239	201	Storey et al. 2007
			CHLARA003	JF433983	183	Storey et al. 2008
			THABNW009	JF433984	201	
	ah3	E1	ASMFTF001(tentative haplotype)	EF535240	166	Storey et al. 2007
			Tonga HB	EF535237	183	Storey et al. 2007
			FSMFSP002 (tentative haplotype)	JF433985	122	
			FSMFSP003	JF433986	178	
			HWIKIP002	JF433987	201	
			PAQANA011	EF535246	192	Storey et al. 2007
			SLB33001	JF433988	225	
			SLBTKP001	JF433989	224	
			SLBTKP002	JF433990	201	
			NIUPKI012 (tentative haplotype)	JF433991	165	
			CHLARA001	EF535241.2	583	Storey et al. 2007
			CHLARA004	JF433992	373	Storey et al. 2008
			ESPALB002	JF433993	201	
			ESPVAL001	JF433994	411	
			ESPLCT001	JF433995	411	
			SPNPRL002	JF433996	198	
			SPNNWL002	JF433997	200	
	ah4		ESPBUZ002	JF433998	199	
	ah5		BOLTAR001	JF433999	366	
			BOLTAR002	JF434000	366	
			BOLTAR003	JF434001	200	
	ah6		PRULOC001	JF434002	201	
	ah7		ESPALB001	JF434003	403	
D	ah9		FSMFSP001	JF434004	162	
			PRUTOR001	JF434005	200	
	ah10		HWIKIP003	JF434006	201	
			HWIKIP004	JF434007	388	
			HWIPLK002	JF434008	394	
			HWIPLR001	JF434009	561	
			HWIPLR002	JF434010	561	
			HWIPLR003	JF434011	166	
			HWIWAI001	JF434012	201	
			HWIWAI002	JF434013	436	
			HWIWAI003	JF434014	201	
			PAQANA004	EF535242	201	Storey et al. 2007
			PAQANA006	EF535243	201	Storey et al. 2007
			PAQANA009	EF535244.2	365	Storey et al. 2007
			PAQANA010	EF535245	200	Storey et al. 2007
			PAQHAN001	EF535247.2	576	Storey et al. 2007
	ah11		HWIKIP005	JF343015	387	
B	ah12		THABNW003	JF434016	201	

* The historic period in Hawai'i begins with first European contact at AD 1778
** The historic period in Easter Island begins in AD 1722
