# Supplementary material for: Investigating the Global Dispersal of Chickens in Prehistory Using Ancient Mitochondrial DNA Signatures
Source: PLoS One. 2012 Jul 25;7(7):e39171. doi: 10.1371/journal.pone.0039171 (PMC3405094; doi:10.1371/journal.pone.0039171)
Supplement: Table S2 — Information relating to the 92 samples acquired for this study. Samples highlighted in blue are those for which mtDNA was amplified. (PDF) [file pone.0039171.s004.pdf]

Supplementary Table S2 Information relating to the 92 samples acquired for this study. Samples highlighted in blue are those for which mtDNA was amplified.

Sample Name	Sample Origin	Provenience Information	Element	Note	Reference
ASMFTF001	Fatu-ma-Futi Site, Samoa	fr: 8797 Ftf DR/AA 2005-10 AS-25-62 TU-46 Tpit 3 (215-220 cmdb	Radius		[1]
ASMFTF002	Fatu-ma-Futi Site, Samoa	fr: 8783 Ftf DR/AA 2005-10 (AS-25-62) STP-17 Spit 2 (110-125 cn	Ulna		[1]
BOLTAR001	Tarapaya, Potosi, Bolivia	FS #169, Level 8	Ulna		[2]
BOLTAR002	Tarapaya, Potosi, Bolivia	FS #169, Level 8	Femur		[2]
BOLTAR003	Tarapaya, Potosi, Bolivia	FS #169, Level 8	Carpometacarpus		[2]
CHLARA001	El Arenal-1 Site, Chile	No 101 EA1/CV3/N6	Humerus		[3, 4]
CHLARA002	El Arenal-1 Site, Chile	No 119 C3/N8	caracoid		[3, 4]
CHLARA003	El Arenal-1 Site, Chile	No 141 C3/N10	Tibiotarsus		[3, 4]
CHLARA004	El Arenal-1 Site, Chile	No 72 C2/N6	Humerus		[3, 4]
ESPALB001	Albarracin (Teruel), Spain	Level UE162	Tibiotarsus		p/c
ESPALB002	Albarracin (Teruel), Spain	Level UE163	Tibiotarsus		p/c
ESPBUZ001	Buzanca (Madrid), Spain	Level VE6875	Tibiotarsus		p/c
ESPBUZ002	Buzanca (Madrid), Spain	Level VE6875 11	Ulna		p/c
ESPLCT001	La Cartuja (Seville), Spain	Level 2.20 - 3.02 Cesspit	Tibiotarsus		p/c
ESPVAL001	Valduno (Asturias), Spain	Tumba 13	Radius		p/c
ESPVAL002	Valduno (Asturias), Spain	Tumba 13	Radius		p/c
FSMKOS001	Kosrae	Bag 43	caracoid		[5]
FSMKOS002	Kosrae	Bag 68	Innominate		[5]
FSMKOS003	Kosrae	Bag 92	Radius		[5]
FSMKOS004	Kosrae	Bag 98	Ulna		[5]
FSMFSP001	FSPO-4 site, Fais	Layer 4	Femur		[6]
FSMFSP002	FSPO-8 site, Fais	Layer 10	Ulna		[6]
FSMFSP003	FSPO-4 site, Fais	Layer 10	Tibiotarsus		[6]
FSMFSP004	FSPO-8 site, Fais	Layer 12	Radius		[6]
FSMFSP005	FSPE-2 site, Fais	Layer 2 (30-40)	Femur	not securely prehistoric, requires direct dating.	[6]
FSMFSP006	FSPO-8 site, Fais	Layer 7 (~130cm)	Femur		[6]
HWIKIP001	Site 1137, Kahikinui, Maui, Hawai'i	Unit J16, Level 1	Unknown shaft fragment		[7]
HWIKIP002	Site 728, Kahikinui, Maui, Hawai'i	Unit Q26, Level 1	Tarsometatarsus		p/c
HWIKIP003	Site 286, Kahikinui, Maui, Hawai'i	Unit TP1, Level 2	Tarsometatarsus		p/c
HWIKIP004	Site 1269, Kahikinui, Maui, Hawai'i	Unit L21, Level 2	Scapula		p/c
HWIKIP005	Site 117, Kahikinui, Maui, Hawai'i	Unit R17, Level 3	Tarsometatarsus		[8]
HWIKUA001	Kualoa, O'ahu	IARII- 866009-027, MSA-4 Firepit Layer III	Unknown shaft fragment		[9]
HWIPLK001	Pelekane Site, Hawai'i	TU-15, Feature 31, Layer VI, Level 1, 65-100/103 cmdbs Cat #71(	Humerus		[10]
HWIPLK002	Pelekane Site, Hawai'i	TU-13 Feature 5, Layer VII, Level 1. 130/144 to 155 cm bs Cat #4	Vertebra		[10]
HWIPLR001	Puu Lanai Ranch Site, Kona, Hawai'i	Site 18497, Layer I	Ulna		[11]
HWIPLR002	Puu Lanai Ranch Site, Kona, Hawai'i	Site 18497, Layer I	Ulna		[11]
HWIPLR003	Puu Lanai Ranch Site, Kona, Hawai'i	Site 18497, Layer I	Fibula		[11]
HWIWAI001	Luala'i, Waimea, Hawai'i	#192	Humerus		p/c
HWIWAI002	Luala'i, Waimea, Hawai'i	#188	Tibiotarsus		p/c
HWIWAI003	Luala'i, Waimea, Hawai'i	#166	Tibiotarsus		p/c
NIUPKI001	Paluki, Niue	433, BB-7	Femur		[12]
NIUPKI002	Paluki, Niue	433, BB-7	Femur		[12]
NIUPKI003	Paluki, Niue	433, BB-7	Femur		[12]
NIUPKI004	Paluki, Niue	433, BB-7	Femur		[12]
NIUPKI005	Paluki, Niue	425, BB-12	Sternum		[12]
NIUPKI006	Paluki, Niue	425, BB-12	Stermn		[12]
NIUPKI007	Paluki, Niue	433, BB-10	Ulna		[12]
NIUPKI008	Paluki, Niue	433, BB-10	Ulna		[12]
NIUPKI009	Paluki, Niue	425, BB-13	Tarsometatarsus		[12]
NIUPKI010	Paluki, Niue	433, BB-20	Tibiotarsus		[12]
NIUPKI011	Paluki, Niue	433, BB-10	Ulna		[12]
NIUPKI012	Paluki, Niue	433 BB-10	Ulna		[12]
PAQANA001	Anakena Site, Easter Island	Layer IX, Level 10, North Bag 316	Tibiotarsus		[13]
PAQANA002	Anakena Site, Easter Island	Layer VIII Level 17, South Bag 235	Carpometacarpus		[13]
PAQANA003	Anakena Site, Easter Island	Layer VI, Level 6, Bag 5071	Tibiotarsus		[13]
PAQANA004	Anakena Site, Easter Island	Layer VIII, Level 7, Bag 5076	Tibiotarsus		[13]
PAQANA005	Anakena Site, Easter Island	Layer VI, Level 3, West Bag 5121	Radius		[13]
PAQANA006	Anakena Site, Easter Island	Layer V, Level 2, North, Unit 5, Bag 5111	Tibiotarsus	burned	[13]
PAQANA007	Anakena Site, Easter Island	Layer VI, Level 6, East 2005, Bag 5071	Tibiotarsus	Sequenced but not included in this paper	[13]
PAQANA008	Anakena site, Easter Island	Layer VIII, Layer 9 Half = North 2004	Radius		[13]
PAQANA009	Anakena Site, Easter Island	Layer IX, Level 10, North	Radius		[13]
PAQANA010	Anakena Site, Easter Island	Layer VII, Level 15, South	Humerus		[13]
PAQANA011	Anakena Site, Easter Island	Layer VIII, Level 18, South	Humerus		[13]

Supplementary Table S2 Information relating to the 92 samples acquired for this study. Samples highlighted in blue are those for which mtDNA was amplified.

Sample Name	Sample Origin	Provenience Information	Element	Note	Reference
PAQANA012	Anakena Site, Easter Island	Layer VIII, Level 126, South	caracoid	Burned	[13]
PAQANA013	Anakena Site, Easter Island	Unit 7, Layer VIII, Level 17, South	Scapula		[13]
PAQHAN001	Hangahahave Site, Easter island	Site 5-79 Feature B4-5 Fallout	Vertebra		[14]
PRULOC001	Locumbilla Winery Site, Peru	L1003.5N/1040E 20NE C	Femur		[15]
PRUTOR001	Torata Alta Site, Peru	Level BL 218, TR.M ON/1E.L5	Tibiotarsus		[2]
SLBS2601	SE-SZ-26-1 , Santa Cruz Island	G14, Layer VI 3.I.78	Unknown shaft fragment		[16]
SLB33001	SE-SZ-33 site Mdailu, Santa Cruz Islan	N7, Layer V	Tarsometatarsus		[16]
SLBANU001	Anuta	TP22 Bone 17-XI-71 T-Box 1352	Humerus		[17]
SLBTKP001	SE-TK-4 site, Tikopia	S15 II, Layer 2	Tibiotarsus		[18]
SLBTKP002	SE-TK-36 site, Tikopia	C2, Layer 3	Tibiotarsus		[18]
SNPNWL002	St. Augustine, Florida	SA26-1, Feature 44, FS#233	Femur		[19]
SPNNWL002	St. Augustine, Florida	SA26-1 Feature 8 FS#160	Tarsometatarsus		[19]
SPNPRL001	Puerto Real, Haiti	Area 35 752N 857E FS#1885	Femur		[20]
SPNPRL002	Puerto Real, Haiti	748N 856E Level 3, FS#2069	Femur		[20]
THABNW001	Ban Non Wat Site, Thailand	BNW Rч 4:5, Bag 76	Tarsometatarsus		[21]
THABNW002	Ban Non Wat Site, Thailand	BNW BNWA 1:3 4	Tarsometatarsus		[21]
THABNW003	Ban Non Wat Site, Thailand	BNW B4 4:5 4	Tibiotarsus		[21]
THABNW005	Ban Non Wat Site, Thailand	BNW'02 As 4:8 2	Ulna		[21]
THABNW006	Ban Non Wat Site, Thailand	BNW As 4:7 7	caracoid		[21]
THABNW007	Ban Non Wat Site, Thailand	BNW 11.2.02 4:11	Humerus		[21]
THABNW008	Ban Non Wat Site, Thailand	BNW 4:17	Unknown shaft fragment		[21]
THABNW009	Ban Non Wat Site, Thailand	BNW A' Squares 2:8 7	Humerus		[21]
THABNW010	Ban Non Wat Site, Thailand	BNW 4:15	Ulna		[21]
Tonga HB	Mele Havea, Tonga	Unit 8, Level 5	Femur		[22]
Tonga TD	Tongoleleka Site, Tonga	Unit 9, Level 5	Femur		[22]
VUTTEO001	Teouma Site, Vanuatu	H6, Area 2, L3, Spit One Id#3687	Tarsometatarsus		[23]
VUTTEO002	Teouma Site, Vanuatu	Area 2, I4, L1 Id#3276	Tarsometatarsus		[23]
VUTTEO003	Teouma Site, Vanuatu	3B.4.6 L3, spit 1, depression feature Id#6.914	Tarsometatarsus		[23]
VUTTEO004	Teouma Site, Vanuatu	J2, 20-30 cm Id#3065	Tarsometatarsus		[23]
VUTTEO005	Teouma Site, Vanuatu	H10, 0-20 cm Id#3041	Tarsometatarsus		[23]
VUTTEO006	Teouma Site, Vanuatu	3A2.8, L3, Top of Burial 50 Id# 8.2295	Humerus		[23]
VUTTEO007	Teouma Site, Vanuatu	3A2.9 L2, Spit 3	Sternum		[23]

p/c = personal communication, publication detailing excavation of these remains forthcoming.
