# Supplementary material for: Investigating the Global Dispersal of Chickens in Prehistory Using Ancient Mitochondrial DNA Signatures
Source: PLoS One. 2012 Jul 25;7(7):e39171. doi: 10.1371/journal.pone.0039171 (PMC3405094; doi:10.1371/journal.pone.0039171)
Supplement: Table S3 — Results of direct radiocarbon dating of some of the samples used in the ancient DNA analysis. Those marked with a single asterisk were published in 2008 [41] and those with two asterisks in 2010 [35] . (DOC) [file pone.0039171.s005.doc]

Supplementary Table S3 Results of direct radiocarbon dating of some of the samples used in the ancient DNA analysis. Those marked with a single asterisk were published in 2008 [24] and those with two asterisks in 2010 [25].

| **Sample Number** | **Lab Number** | **Radiocarbon Age (BP)** | **^13^C‰** | **^15^N‰** | **^34^S‰** | **C:N** |
| --- | --- | --- | --- | --- | --- | --- |
| CHLARA001* | NZA26115 | 622±35 | -20.9 |  |  |  |
| CHLARA003* | NZA28271 | 510±30 | -19.9 | 15.1 | 2.16 | 3.2 |
| CHLARA004* | NZA28272 | 506±30 | -19.3 | 16.1 |  | 3.2 |
| ESPLCT001 | NZA29456 | 315±35 | -18.8 |  |  |  |
| ESPALB001 | NZA29450 | 930±50 | -19.2 |  |  |  |
| VUTTEO003** | NZA29453 | 2757±30 | -20.5 | 12.36 |  | 3.4 |
| VUTTEO006** | Wk 25198 | 2944±30 | -18.5 | 9.8 |  | 3.4 |
