# Supplementary material for: Investigating the Global Dispersal of Chickens in Prehistory Using Ancient Mitochondrial DNA Signatures
Source: PLoS One. 2012 Jul 25;7(7):e39171. doi: 10.1371/journal.pone.0039171 (PMC3405094; doi:10.1371/journal.pone.0039171)
Supplement: Table S4 — Primers employed in the amplification of overlapping fragments of short template DNA. (DOC) [file pone.0039171.s006.doc]

Supplementary Table S4 Primers employed in the amplification of overlapping fragments of short template DNA.

| **Name of Primer** | **Direction** | **Sequence** |
| --- | --- | --- |
| GG144F | Forward | 5’ ACCCATTATATGTATACGGGCATTAA |
| GG313R | Reverse | 5’aaccattcatagttaggagacttgtt |
| GG218F | Forward | 5’CATTCACCCTCCCCATAGACAG |
| G387R | Reverse | 5’ CGAGCATAACCAAATGGGTTAGA |
