# Supplementary material for: Investigating the Global Dispersal of Chickens in Prehistory Using Ancient Mitochondrial DNA Signatures
Source: PLoS One. 2012 Jul 25;7(7):e39171. doi: 10.1371/journal.pone.0039171 (PMC3405094; doi:10.1371/journal.pone.0039171)
Supplement: Table S5 — Number of uniquely derived amplicons for each sample published for the first time in this paper. (DOC) [file pone.0039171.s007.doc]

Supplementary Table S5 Number of uniquely derived amplicons for each sample published for the first time in this paper.

|  | **144-313** | **144-387** | **218-313** |
| --- | --- | --- | --- |
| **Sample Name** |  |  |  |
| BOLTAR001 |  | **6** |  |
| BOLTAR002 |  | **2** |  |
| BOLTAR003 |  | **4** |  |
| ESPALB001 |  | **2** |  |
| ESPALB002 |  | **4** |  |
| ESPBUZ002 |  | **2** |  |
| ESPLCT001 |  | **2** |  |
| ESPVAL001 |  | **2** |  |
| FSMFSP001 | **2** |  | **4** |
| FSMFSP002 | **2** |  | **3** |
| FSMFSP003 |  | **2** |  |
| HWIKIP002 |  | **3** |  |
| HWIKIP003 |  | **3** |  |
| HWIKIP004 |  | **4** |  |
| HWIKIP005 | **2** | **2** |  |
| HWIPLK002 |  | **2** |  |
| HWIPLR001 |  | **3** |  |
| HWIPLR002 |  | **2** |  |
| HWIPLR003 |  | **2** |  |
| HWIWAI001 |  | **4** |  |
| HWIWAI002 |  | **4** |  |
| HWIWAI003 |  | **4** |  |
| NIUPKI012 |  |  | **2** |
| PRULOC001 |  | **2** |  |
| PRUTOR001 |  | **2** |  |
| SLBS33001 |  | **2** |  |
| SLBTKP001 |  | **2** |  |
| SLBTKP002 |  | **2** |  |
| SPNNWL002 |  | **2** |  |
| SPNPRL002 |  | **2** | **1** |
| THABCH003 |  | **2** |  |
| THABCH009 |  | **2** | **2** |
