# Supplementary material for: Investigating the Global Dispersal of Chickens in Prehistory Using Ancient Mitochondrial DNA Signatures
Source: PLoS One. 2012 Jul 25;7(7):e39171. doi: 10.1371/journal.pone.0039171 (PMC3405094; doi:10.1371/journal.pone.0039171)
Supplement: Citations S1 — Supplementary Citations. (DOC) [file pone.0039171.s008.doc]

# SI Citations

1. Addison DJ, Walter G, & Morrison A (2008) The Initial Investigation of Fatu-ma-Futi: An Ancient Coastal Village Site, Tutuila Island, Territory of American Samoa. New Zeal J Archaeol 29: 89-107.
2. deFrance SD (1996) Iberian Foodways in the Moquegua and Torata Valleys of Southern Peru. Hist Archaeol 30(3): 20-48.
3. Storey AA, Quiroz D, Beavan NR, Matisoo-Smith E (2011) Pre-Columbian chickens of the Americas: A critical review of the hypotheses and evidence for their origins. Rapa Nui J 25: 5-19.
4. Contreras L, Quiroz D, Sanchez M, & Caballero C (2005) Ceramios, maíces y ranas… Un campamento El Vergel en las costas de Arauco. Actas del XVI Congreso Nacional de Arqueología Chilena. Concepcion: Escaparte. pp 357-367.
5. Athens JS (1995) Landscape archaeology : prehistoric settlement, subsistence and environment of Kosrae, Eastern Caroline Islands, Micronesia : archaeological data recovery investigations for the Kosrae Wastewater Project, Contract C22273. Honolulu: International Archaeological Research Institute. 474p.
6. Intoh M (2008) Ongoing archaeological research on Fais Island, Micronesia. Asian Perspectives 47: 121-138.
7. Jones O'Day S (2001) Excavations at the Kipapa Rockshelter, Kahikinui, Maui, Hawai'i. Asian Perspectives 40(2): 279-305.
8. Kirch PV (1997) N'a mea kahiko o Kahikinui : studies in the archaeology of Kahikinui, Maui. Berkeley, CA: Oceanic Archaeology Laboratory Archaeological Research Facility University of California Berkeley. 81p.
9. Carson MT & Athens JS (2006) Archaeological Monitoring and Data Recovery at Kualoa Regional Park, Kualoa Ahupua'a, Ko'olaupoko district, O'ahu Island, Hawai'i.: Report prepared for Haitsuka Brothers, Ltd., Honolulu. Honolulu: International Archaeological Research Institute, Inc.
10. Dye TS, Maly K, & Athens JS (2002) Supplemental Research to Support an Archaeological Inventory Survey of Puu Lani Ranch Phase II, Pu'u Anahulu, Kona, Hawai'i. Honolulu: International Archaeological Research Inc.
11. Walter R & Anderson A (2002) The Archaeology of Niue Island, West Polynesia. Honolulu.: Bishop Museum Press.
12. Hunt TL & Lipo CP (2006) Late Colonization of Easter Island. Science 311: 1603-1606.
13. Ramírez JM (2005) El crematorio de Hanga Hahave, Rapa Nui. In: Stevenson, CM, Ramírez Aliaga JM, Morin FJ, & Barbacci N editors. The Reñaca Papers. VI International Conference on Easter Island and the Pacific: VI Congreso Internacional sobre Rapa Nui y el Pacifico. Los Osos, California : The Easter Island Foundation. pp 449-455.
14. deFrance SD (2010) Paleopathology and health of native and introduced animals on Southern Peruvian and Bolivian Spanish Colonial sites. Int Journal Osteoarchaeol 20(5): 508-524.
15. McCoy PC & Cleghorn PC (1988) Archaeological excavations in Santa Cruz (Nendo), Southeast Solomon Islands: Summary report*.* Archaeology in Oceania 23(3): 104-115.
16. Kirch PV & Rosendahl PH (1973) Archaeological Investigation of Anuta. In Yen DE, Gordon J, editors. Anuta : a Polynesian outlier in the Solomon Islands. Honolulu: Department of Anthropology Bernice Pauahi Bishop Museum. Pp. 25-108.
17. Kirch PV & Yen DE (1982) Tikopia: the Prehistory and Ecology of a Polynesian Outlier. Honolulu: Bishop Museum Bulletin 238..
18. Deagan K (1983) Spanish St. Augustine: The Archaeology of a Colonial Creole Community. New York: Academic Press.
19. Reitz EJ & McEwan BG (1995) Animals, Environment, and the Spanish Diet at Puerto Real. In Deagan K , editor. Puerto Real: The Archaeology of a Sixteenth-Century Spanish Town in Hispaniola. Gainesville: University Press of Florida. Pp. 287-334.
20. Higham C & Higham TFG (2009) A new chronological framwork for prehistoric Southeast Asia, based on a Bayesian model from Ban Non Wat. Antiquity 83: 125-144.
21. Burley DV (1997) Report of the 1997 Lapita Project, Ha'apai Islands, Kingdom of Tonga. Nuku’alofa: Unpublished Report on file, Prime Ministers Office. Available online at <http://www.sfu.ca/archaeology-old/museum/tonga/toc.html>
22. Bedford S, Spriggs M, Buckley HR, Valentin F, & Regenvanu R (2009) The Teouma Lapita Site, South Efate, Vanuatu: A Summary of Three Field Seasons (2004-2006). *Lapita: Ancestors and Descendants*, eds Sheppard P, Thomas T, & Summerhayes G (New Zealand Archaeological Association, Auckland), pp 215-234.
23. Storey AA, Quiroz D, Ramirez JM, Beavan-Athfield NR, Addison DJ, et al. (2008) Pre-Columbian chickens, dates, isotopes and mtDNA. Proc Natl Acad Sci USA 105: E99.
24. Storey AA, Spriggs M, Bedford S, Hawkins SC, Robins JH, et al. (2010) Mitochondrial DNA from 3000-year old chickens at the Teouma Site, Vanuatu. J Archaeol Sci 37: 2459-2468.
25. Liu Y-P, Wu G-S, Yao YG, Miao Y-W, Luikart G, et al. (2006) Multiple maternal origins of chickens: Out of the Asian jungles. Mol Phylogenet Evol 38: 12-19.
